# Dr. Eugene Beach

**Published:** 1909-08

**Authors:** 


### OBITUARY

Dr. Eugene Beach, of Gloversville, State Medical Examiner, died Wednesday at midnight, July 7, 1909. Dr. Beach was seized with cerebral apoplexy early in the day, while engaged in the performance of his official duties, relating to the June examination papers. He lived only a few hours after the seizure.

Dr. Beach had been a member of the State Board of Medical Examiners since the law went into operation in 1891. He was a man of distinguished ability, not only in his profession, but in the general walks of life. He had been Mayor of his city, being chosen on a reform platform, and demonstrated his executive capacity in that office to the satisfaction of the people without regard to political preference.

As a member of the State Board of Medical Examiners, Dr. Beach performed his duties with singular fidelity and impartiality. To say that Dr. Beach was beloved by every member of the Board is not exaggeration. His loss is deeply felt not only by every member of the board, but by all who knew him.

